# A fenestrated persistent primitive hypoglossal artery harboring a ruptured aneurysm: A case report

**DOI:** 10.1097/MD.0000000000026904

**Published:** 2021-08-13

**Authors:** Sen He, Ming-Li Wei, Fei Xie, Seidu A. Richard

**Affiliations:** a Department of Neurosurgery, The First People's Hospital of Ziyang City, No.66 Rende West Road, Ziyang, PR China; b Department of Respiratory, The First People's Hospital of Ziyang City, No.66 Rende West Road, Ziyang, PR China; c Department of Medicine, Princefield University, Ho-Volta Region, Ghana West Africa.

**Keywords:** aneurysms, artery, embolization fenestration, hypoglossal, primitive

## Abstract

**Rationale:**

Persistent primitive hypoglossal artery (PPHA) is a rare and permanent carotid-vertebrobasilar anastomoses. Patients with PPHA usually have higher changes of developing intracranial aneurysms due the high intracranial hemodynamics. Although cases of PPHA alone and PPHA with aneurysms have been reported in literature, cases of fenestrated PPHA harboring a ruptured aneurysm have seldomly be reported in literature. We present a rare occurrence of a fenestrated PPHA harboring a reputed aneurysm.

**Patients concerns:**

A 43-year-old woman was presented with a sudden-onset severe headache and nausea.

**Diagnosis:**

Computerized tomography scan showed third, fourth, and bilateral ventricular hemorrhages. Computed tomographic angiogram showed a PPHA with fenestration malformation and a cystic protrusion consistent with an aneurysm.

**Intervention:**

The patient underwent a successful stent-assisted coil embolization via the trans-arterial route under general anesthesia.

**Outcomes:**

Two years follow-up revealed no recurrence of her symptomatology and she is currently well and go about her normal daily life.

**Conclusion:**

Fenestrated PPHAs harboring aneurysms may be more prone to rupture because of the fenestration and connective tissue weakness of the artery as well as changes in hemodynamics of the already malformed and weak artery.

## 1 Introduction

Persistent primitive hypoglossal artery (PPHA) is a rare and permanent carotid-vertebrobasilar anastomoses with an incidence rate of about 0.02% to 0.26%.^[1–3]^ The PPHA mostly originates from the internal carotid artery (ICA) and rarely from the external carotid artery.^[4]^ Patients with PPHA usually have higher changes of developing intracranial aneurysms due the high intracranial hemodynamics.^[5–8]^ Most often than not, the aneurysms are often found on other arteries and not the parent artery with the PPHA.^[8,9]^ However, a few cases of commitment occurrences of aneurysms on the parent PPHA artery have been reported.^[8,9]^

Computed tomographic angiography (CTA) is usually the gold standard radiological modality for the diagnosis as well as accurate evaluation of the persistent anastomoses.^[9]^ Surgical evaluation as well as clipping of the aneurysms on the parent PPHA in the posterior circulation is often very difficult.^[9]^ Thus, endovascular coiling is the currently the safest and easier treatment option for acutely ruptured intracranial arterial aneurysms with decreased morbidity as well as morbidity in complex PPHA harboring aneurysms as compared with open surgery.^[3–9]^ We report a rare case of a fenestrated PPHA harboring a ruptured aneurysm which we successfully treated with stent-assisted coil embolization. Although cases of PPHA alone and PHHA with aneurysms have been reported in literature, cases of fenestrated PPHA harboring a ruptured aneurysm have seldomly be reported in literature.

## 2 Case report

A 43-year-old woman was admitted at our neurosurgery department due to sudden-onset of severe headache and nausea. Her past medical history was unremarkable. Cranial nerves examination revealed no focal neurological findings. General physical examination did not yield much. Routine laboratory investigations were grossly normal. Also, routine chest x-ray and electrocardiogram did not show any abnormalities.

Head computerized tomography (CT) scan showed third, fourth, and bilateral ventricular hemorrhages (Fig. 1 A and B). Also, CT scan showed a PPHA originated from the left ICA at the plane of the C1 and C2 vertebral bodies (Fig. 1 C) and entered the skull through the hypoglossal canal (Fig. 1 D). CTA further confirmed a PPHA with a fenestrated malformation and a cystic protrusion consistent with an aneurysm (Fig. 1 E). Also, the bilateral posterior communicating arteries were absent with very tin vertebral arteries (Fig. 1 F). Digital subtraction angiography (DSA) confirmed that, the bilateral posterior inferior cerebellar arteries originated from the fenestrated PPHA which also harbored the ruptured arterial aneurysm (Fig. 1 H). The blood supply of PPHA was from the ipsilateral ICA (Fig. 1 G).

**Figure 1:**
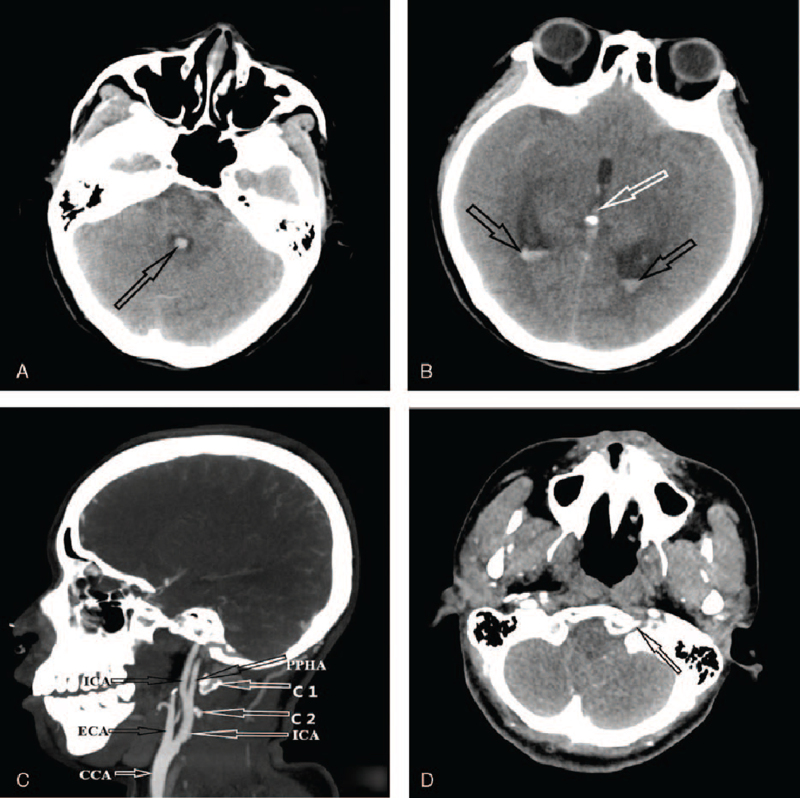
A–D: Are preoperative CT scan, images showing hemorrhage and a PPHA. A: Is a CT scan showing fourth ventricular hemorrhage. B: Is a CT scan showing third ventricular and bilateral ventricular hemorrhages. C: Is a CT scan showing a PPHA originating from the internal carotid artery (ICA) at the plane of the C1 and C2 vertebral bodies. D: Is a CT scan showing a PPHA entering the skull through the hypoglossal canal. E–H: Are preoperative CTA and DSA images showing a fenestrated malformation with a cystic protrusion on the lower left side. E: Is a CTA showing a fenestration of the PPHA after entering the skull with a ruptured aneurysm. The bilateral posterior communicating arteries are absent. F: Is a DSA image showing very tine bilateral vertebral arteries throughout their course and do not provide blood supply to the basilar artery. G: Is a DSA image showing the posterior circulation. The blood supply of PPHA comes from the ipsilateral internal carotid artery. H: Is a DSA image showing a PPHA, an aneurysm and a fenestrated malformation. CT = computerized tomography, CTA = computed tomographic angiography, DSA = digital subtraction angiography, PPHA = persistent primitive hypoglossal artery.

**Figure 1 (Continued):**
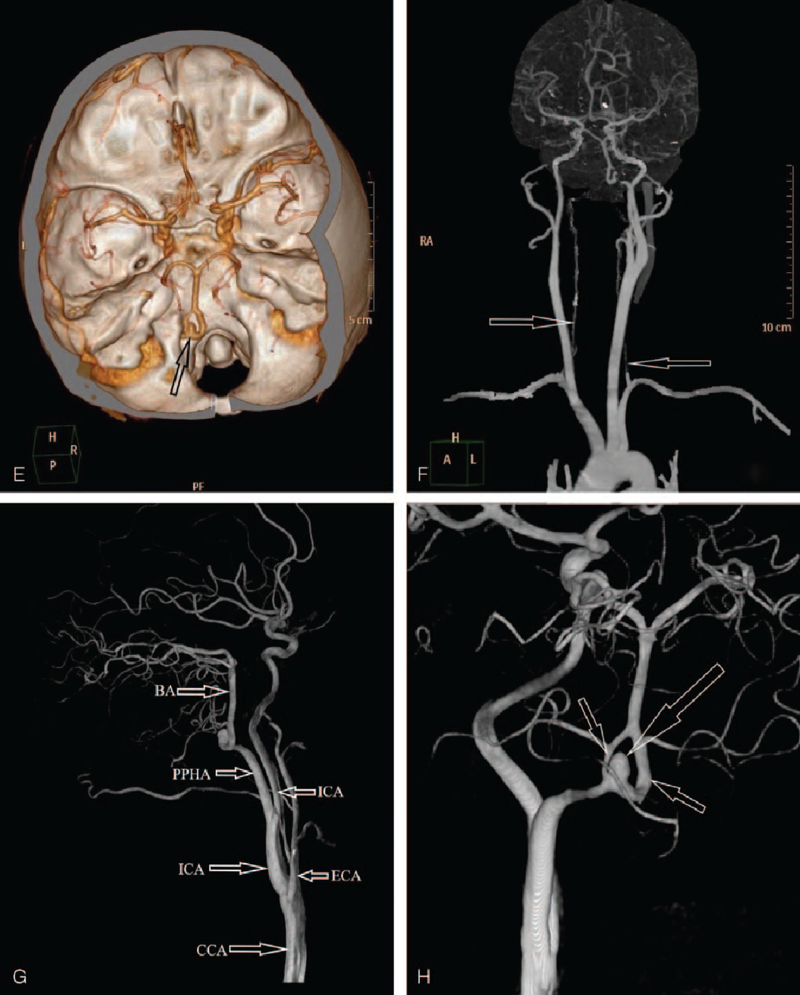
A–D: Are preoperative CT scan, images showing hemorrhage and a PPHA. A: Is a CT scan showing fourth ventricular hemorrhage. B: Is a CT scan showing third ventricular and bilateral ventricular hemorrhages. C: Is a CT scan showing a PPHA originating from the internal carotid artery (ICA) at the plane of the C1 and C2 vertebral bodies. D: Is a CT scan showing a PPHA entering the skull through the hypoglossal canal. E–H: Are preoperative CTA and DSA images showing a fenestrated malformation with a cystic protrusion on the lower left side. E: Is a CTA showing a fenestration of the PPHA after entering the skull with a ruptured aneurysm. The bilateral posterior communicating arteries are absent. F: Is a DSA image showing very tine bilateral vertebral arteries throughout their course and do not provide blood supply to the basilar artery. G: Is a DSA image showing the posterior circulation. The blood supply of PPHA comes from the ipsilateral internal carotid artery. H: Is a DSA image showing a PPHA, an aneurysm and a fenestrated malformation. CT = computerized tomography, CTA = computed tomographic angiography, DSA = digital subtraction angiography, PPHA = persistent primitive hypoglossal artery.

**Figure 2:**
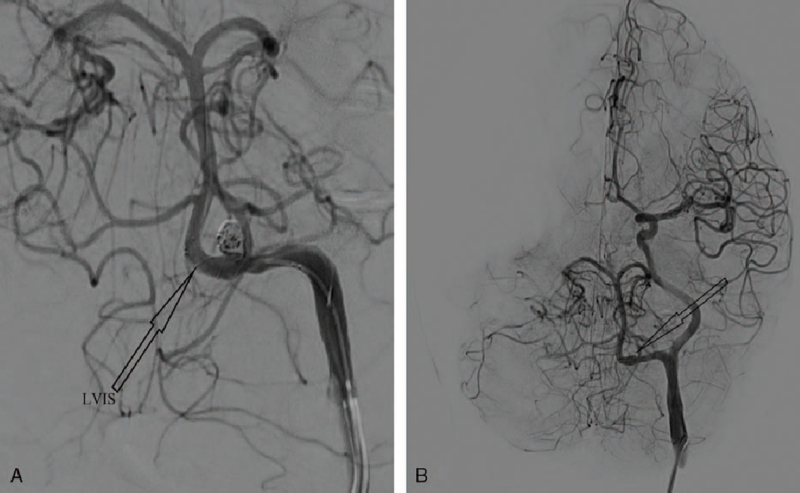
Are intraoperative as well as postoperative DSA images. A, Is an intraoperative DSA image showing a LVIS stent-assisted coil embolization in-situ. B: Is 6 months postoperative DSA image showing complete obliteration of the aneurysm with no stenosis in the PPHA. DSA = digital subtraction angiography, LVIS = low-profile visible intraluminal support, PPHA = persistent primitive hypoglossal artery.

The patient underwent stent-assisted coil embolization via the trans-arterial route under general anesthesia. She was given oral aspirin and clopidogrel (300 mg each) on the day of the surgery and 40 mg heparin (approximately 2/3 of her 60 kg weight) intraoperatively. Intraoperatively, we observed a ruptured aneurysm in a fenestrated PPHA on the left ICA. The aneurysm was located along the blood flow at the proximal bifurcation of the fenestration. The neck of the aneurysm was relatively wide. The aneurysm was successfully embolized with low-profile visible intraluminal support (LVIS) stent-assisted coils (Microvention, Tustin, CA) (Fig. 2A). Seven days after the surgery, she was discharged from the hospital on 75 mg clopidogrel daily for 3 months and 100 mg aspirin daily for 6 months.

There were no postoperative complications and no neurological deficits. First 6 months follow-up visit revealed resolution of her symptomatology. A repeated DSA confirmed complete obliteration of the aneurysm with no stenosis in the PPHA (Fig. 2B). Two years follow-up revealed no recurrence of her symptomatology and she is currently well and go about her normal daily life.

## 3 Discussion

The occurrence of aneurysms on the normal hypoglossal artery is very rare.^[10,11]^ Nevertheless, in the presence of a PPHA, the probability of an aneurysm to occur rises to about 26%.^[10,11]^ The PPHA often triggers changes in the hemodynamics of the carotid as well as vertebrobasilar arteries resulting in the formation of the aneurysms.^[4,12,13]^ PPHAs are commonly seen in women as compared with men and they often occurs on the left side.^[4,6,14]^ PPHAs often originate from the ICA, external carotid artery, or both sides.^[15–17]^ Apart from aneurysm, this anomaly is also associated with arteriovenous malformations, moyamoya disease, ischemic stroke, atherosclerosis, subarachnoid hemorrhage as well as tumors.^[4,12,13]^ Although cases of PPHA alone and PPHA with aneurysms have been reported in literature, cases of fenestrated PPHA harboring a ruptured aneurysm have seldomly be reported in literature.

Primitive arteries like the trigeminal artery, auricular artery, hypoglossal artery, as well as anterior atlantoaxial intersegmental artery form the transient vascular anastomotic pathways from the superior to the inferior parts between the primitive carotid and vertebrobasilar arteries during the development of the embryonic craniocerebral circulation.^[9,14,16]^ These vascular anastomoses gradually degenerate and disappear.^[8,16]^ If they do not degenerate and persist into birth or even adulthood, they can become permanent carotid-vertebrobasilar anastomoses.^[9,14,16]^ Among the 4 undegenerated vascular anastomoses, PPHA is the second most common.^[8,16]^

PPHA normally originates from the cervical segment of the ICA, at the plane of the C1–C3 vertebral bodies.^[4,9,14,16]^ After entering the skull through the hypoglossal canal and anastomoses with the basilar artery, PPHA becomes the only artery to supply the basilar artery.^[8,9,14,16]^ Its presence commonly concurs with the dysplasia of the posterior communicating arteries as well as vertebral arteries.^[9,14,16]^ The presentation of our patient was fully compatible with these descriptions.

Most patients with PPHA were identified incidentally.^[1,2,16,17]^ Nevertheless, some patients presented with hypoglossal nerve palsy, glossopharyngeal neuralgia, or aneurysm rupture.^[14,16,18]^ The cardinal presentation in our case was sudden-onset severe headache and nausea as a result of the intracranial hemorrhage. Although PPHA increased the risk of intracranial aneurysm,^[1,7,14]^ very few aneurysms have been reported in PPHA itself.^[4,9,16]^ A concurrent fenestrated malformation is even rarer. CT scan is usually the initial radiological evaluation method^[9]^ because, it capable of detecting hemorrhage and as the PPHA as it enters the skull as seen in our case. Also, in our case, CT scan revealed third, fourth, and bilateral ventricular hemorrhages. CTA offers accurate anatomical localization because of its ability to display the vessel entering the expanded hypoglossal canal.^[9]^ Furthermore, it has the advantage of multiplanar validation especially when using 3D reconstruction.^[9]^

Magnetic resonance imaging and magnetic resonance angiography (MRA), to be specific, are capable of adequately displaying the PPHA without any contrast medium.^[9]^ MRA is also capable of revealing the complete carotid-basilar anastomosis without superimposition of other vessels when the maximum intensity projection program is used.^[9]^ We did not perform magnetic resonance imaging or MRA in our case because the CT, CTA, and DSA were adequate in establishing the definitive diagnosis. CTA with 3D reconstructions is often the most precise preoperative modality because, it capable of showing the location, diameters, neck as well as morphology the PPHA.^[9,19]^

Surgical treatment via either wrapping or clipping of the aneurysm was the initial treatment for PPHA with aneurysms.^[20,21]^ However, surgical approach is always challenging for these aneurysms in the posterior circulation.^[9]^ Surgical unreachability, unsuccessfulness of clipping, wide or absent aneurysm neck, and a medical illness impeding craniotomy were the initial potential indications of endovascular intervention for the treatment of PPHA.^[9,22]^ In our case, the bilateral posterior inferior cerebellar arteries originated from the fenestrated malformed vessels. The aneurysm was located along the blood flow at the proximal bifurcation of the fenestration, which suggested involvement of hemodynamic forces during the development of aneurysm. The neck of the aneurysm was relatively wide. Thus, we selected LVIS stent-assisted embolization, which had a high metal coverage, in order to reduce the recurrence of the aneurysm and avoid the risk of PPHA stenosis.

LVIS device is a new brand of intracranial stent in recent years.^[23,24]^ These devices are flexible, braided microstent made purposely for the stent-assisted coiling of wide-necked intracranial aneurysms.^[24,25]^ They have demonstrated to be effective as well as efficient embolization treatment option for intracranial aneurysms.^[23,24]^ They have good compliance as well as vascular accesses.^[24]^

## 4 Conclusion

Fenestrated PPHAs harboring ruptured aneurysms have seldomly been reported in literature. Fenestrated PPHAs harboring aneurysms may be more prone to rupture because of the fenestration and connective tissue weakness of the artery as well as changes in the hemodynamics of the already malformed and weak artery.

## Author contributions

All authors contributed toward data collection, drafting, and critically revision of the paper and agree to be accountable for all aspects of the work. Seidu A. Richard wrote the final paper.

**Conceptualization:** Sen He, Ming-Li Wei, Fei Xie, Seidu A. Richard.

**Data curation:** Sen He, Ming-Li Wei, Fei Xie, Seidu A. Richard.

**Formal analysis:** Sen He, Ming-Li Wei, Fei Xie, Seidu A. Richard.

**Methodology:** Sen He, Ming-Li Wei, Fei Xie, Seidu A. Richard.

**Resources:** Sen He, Ming-Li Wei, Fei Xie.

**Supervision:** Fei Xie.

**Writing – original draft:** Seidu A. Richard.

**Writing – review & editing:** Sen He, Ming-Li Wei, Fei Xie, Seidu A. Richard.
